# Temporal multiomics gene expression data of human embryonic stem cell-derived cardiomyocyte differentiation

**DOI:** 10.1038/s41597-025-05655-9

**Published:** 2025-07-28

**Authors:** Abdurrahman Keskin, Hani J. Shayya, Dario Sirabella, Achchhe Patel, Barbara Corneo, Marko Jovanovic

**Affiliations:** 1 Department of Biological Sciences, Columbia University, New York, NY 10027 USA; 2 Mortimer B. Zuckerman Mind, Brain and Behavior Institute, Columbia University, New York, NY 10027 USA; 3 Columbia Stem Cell Initiative, Stem Cell Core, Columbia University Irving Medical Center, New York, NY 10032 USA

**Keywords:** Proteomics, Embryonic stem cells, Transcriptomics

## Abstract

Human embryonic stem cells (hESCs) serve as a valuable *in vitro* model for studying early human developmental processes due to their ability to differentiate into all three germ layers. Here, we present a comprehensive multi-omics dataset generated by differentiating hESCs into cardiomyocytes via the mesodermal lineage, collecting samples at 10 distinct time points. We measured mRNA levels by mRNA sequencing (mRNA-seq), translation levels by ribosome profiling (Ribo-seq), and protein levels by quantitative mass spectrometry-based proteomics. Technical validation confirmed high quality and reproducibility across all datasets, with strong correlations between replicates. This extensive dataset provides critical insights into the complex regulatory mechanisms of cardiomyocyte differentiation and serves as a valuable resource for the research community, aiding in the exploration of mammalian development and gene regulation.

## Background & Summary

Embryonic development is a highly intricate process that, despite significant insights gained over the last decades through molecular and systems-level approaches, as well as advancements in genomic technologies, is still not fully understood. Gaining insight into how unique gene expression patterns are established and maintained in each cell type during embryogenesis is essential for advancing our knowledge of developmental biology. However, investigating the gene regulatory mechanisms that govern early embryonic development frequently requires the use of embryonic cell types, which are currently challenging or impractical to acquire in humans. Human embryonic stem cells (hESCs)^1^ are pluripotent cells capable of differentiating into all three germ layers of a developing embryo, making them an ideal *in vitro* model for investigating early human developmental processes. The differentiation of hESCs into various cell types and tissues can mimic the early stages of embryonic development and provide insights into the molecular events that drive cell fate decisions.

**Fig. 1:**
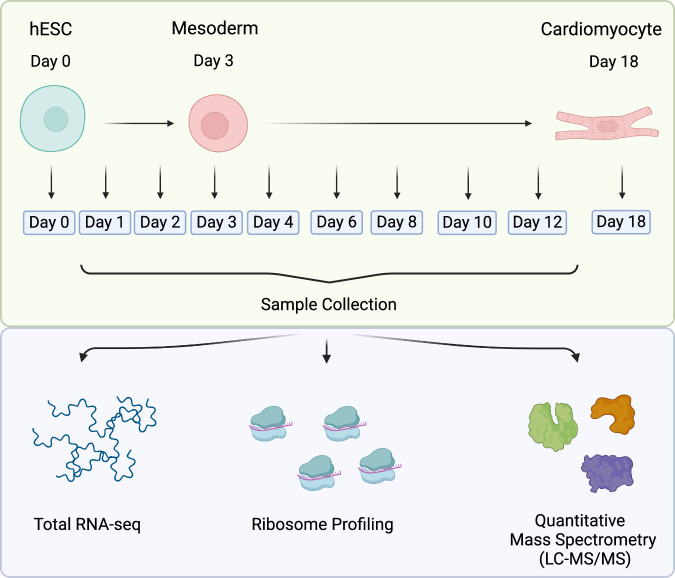
Experimental design and multi-omics data generation. hESCs were differentiated into the mesoderm germ layer and subsequently into cardiomyocytes. Samples were collected at 10 different time points during the time-course of hESC differentiation, and matched samples were measured for RNA, translation, and protein levels by RNA-seq, Ribosome profiling, and mass spectrometry, respectively. Created with BioRender.com.

To gain a deeper understanding of the gene regulatory mechanisms that drive embryonic development, we conducted simultaneous genome-wide measurements of mRNA, translation, and protein levels during hESC differentiation. This involved differentiating hESCs into the three distinct germ layers—endoderm, mesoderm, and ectoderm—and subsequently further differentiating them into polyhormonal cells, cardiomyocytes, and motor neurons, respectively. In this study, we specifically focus on the differentiation process from hESCs to cardiomyocytes via the mesodermal lineage. We collected samples at 10 distinct time points during cardiomyocyte differentiation, which allows us to track the differentiation of cells over time, providing valuable insights into the temporal regulation of gene expression. We measured mRNA levels by mRNA sequencing (mRNA-seq), translation level proxies by ribosome profiling (Ribo-seq), and protein levels by quantitative mass spectrometry-based proteomics on the matched samples (Fig. 1). The differentiation and subsequent measurements were conducted in duplicates. To our knowledge, the extensive temporal resolution and multi-omics approach of our study represent one of the most comprehensive datasets in the field, providing critical insights into the complex regulatory mechanisms of cardiomyocyte differentiation.

We believe our system-level approach integrating mRNA, translation, and protein time-course measurements is essential for comprehensively understanding gene regulatory mechanisms in developmental systems. While mRNA levels indicate gene transcription activity, they do not always accurately reflect the levels of the corresponding proteins due to regulation at the post-transcriptional level^2–4^. Protein levels, which result from mRNA abundance, translational efficiency, and protein stability, are the final effectors of cellular functions. Therefore, integrating these measurements provides a holistic view of gene regulation during embryonic development and differentiation.

The deep dataset we generated can uncover novel insights into mammalian development. By comparing RNA, protein, and translation levels across all three differentiations, we can identify commonalities and key differences that underpin cell differentiation and organogenesis. This dataset also allows us to generate new hypotheses, potentially driving future research in developmental and molecular biology. Furthermore, integrating these data with other genetic and epigenetic datasets will enable a more comprehensive understanding of the complex processes that drive mammalian development. Overall, the deep data set generated by our study represents a valuable resource for researchers seeking to explore the intricacies of gene expression and regulation during mammalian development.

## Methods

### Mesoderm - Cardiomyocyte (CM) differentiation

Cardiomyocyte differentiation was performed as described previously^5^ with slight modifications. hESCs (RUES2 cell line) were maintained and expanded in Matrigel-coated plates with mTeSR Plus medium (StemCell Technologies). Cells were grown to confluency (90%) before starting the differentiation. On Day 0 (start of differentiation), hESCs were treated with 1 ml/well (6-well plates) Collagenase B (1 mg/ml) and DNase (10 µl/ml) for 30 min to form embryoid bodies (EBs). After Collagenase B digestion, the aggregates were allowed to settle in a tube for 10–20 min and resuspended by gentle pipetting in the CM differentiation medium consisting of RPMI 1640 with ascorbic acid (50 µg/ml), L-glutamine (2 mM/L), and monothioglycerol (MTG) (4 × 10^−4^ M). Differentiation medium was also supplemented with BMP4 (2 ng/ml) and Rock Inhibitor (10 µM), and the EBs were cultured in low attachment 6-well plates (Corning) at 37 °C in a humidified incubator with 5% CO_2_, 5% O_2_, and 90% N_2_. On Day 1, the medium was changed to the differentiation medium supplemented with BMP4 (20 ng/ml), Activin A (20 ng/ml), bFGF (5 ng/ml), and Rock Inhibitor (10 µM) for primitive streak/germ layer induction. On Day 3, the EBs were harvested and washed with DMEM to remove all residual cytokines, and the medium was changed to the differentiation medium supplemented with XAV (5 µM) and VEGF (5 ng/ml) to induce cardiac mesoderm. On Day 5, the medium was changed to the differentiation medium supplemented with VEGF (5 ng/ml). On Day 7, the EBs were fed with the medium same as on Day 5. After Day 10, the medium was changed to the differentiation medium without any supplements every 3 to 4 days.

### Sample collection

Sample collection was done on Day 0, Day 1, Day 2, Day 3, Day 4, Day 6, Day 8, Day 10, Day 12, and Day 18, a total of 10 time points. RNA-seq and Ribosome Profiling samples were resuspended in the polysome lysis buffer consisting of 20 mM Tris pH = 7.4, 250 mM NaCl, 15 mM MgCl_2,_ 100 µg/ml cycloheximide, 1 mM dithiothreitol (DTT) and 0.5% Triton X-100. The polysome lysis buffer was supplemented with 10x protease inhibitor, 0.04 U/µl TurboDNase, and 0.4 U/µl RNasin. Cells were not pretreated with cycloheximide. Proteomics samples were resuspended in the urea buffer composed of 8 M Urea, 75 mM NaCl, 50 mM Tris pH = 8.0, and 1 mM EDTA

### Cell line authentication and quality control

We utilized the RUES2 hESC line, which was derived at Rockefeller University. Detailed information regarding the derivation and characterization of this cell line can be found through the following link: https://xenopus.rockefeller.edu/stemcell/rues2. Additional characterization data are available on the hPSCreg website, where this cell line is registered: https://hpscreg.eu/cell-line/RUESe002-A.

The RUES2 cell line has been authenticated and tested for integrity at Columbia Stem Cell Core, as well. Karyotyping was performed at passages 18, 34, and 39, confirming a normal human female karyotype. Furthermore, mycoplasma contamination testing was conducted multiple times during the cell culture process, with all results indicating no mycoplasma contamination.

### RNA-seq library construction

Homogenates were cleared by centrifugation at 13,000 × *g* for 10 min at 4 °C. Total RNA was isolated from the collected supernatant by a QIAGEN RNeasy kit, and the quality of total RNA was checked by a Bioanalyzer (Agilent). Ribosomal RNA was depleted by the NEBNext rRNA depletion kit (Human/Mouse/Rat) according to the manufacturer’s instructions. Strand-specific sequencing libraries were generated from rRNA-depleted total RNA samples by using NEBNext Ultra II Directional RNA Library Prep Kit for Illumina following the manufacturer’s instructions. The libraries were quantified using a Qubit dsDNA HS kit (Thermo Fisher Scientific), and the library quality was assessed by a Bioanalyzer (Agilent). Paired-end sequencing was performed using an Illumina NextSeq500 desktop sequencer with a read length of 75 bases.

### Ligation-free ribosome profiling

Ribosome profiling was performed as described previously^6^ with slight modifications. Briefly, homogenates were clarified by centrifugation at 13,000 × *g* for 10 min at 4 °C. Clarified cell lysates were treated with *E. coli* RNase I (Ambion) and incubated for 30 min at room temperature. Ribosome-protected fragments were pelleted in 50% sucrose solution using an Ultracentrifuge (Beckman, with TLA-100.3 rotor) at 70,000 rpm for 3.5 hours at 4 °C and isolated by Trizol extraction. Ribosome-protected fragments in ∼28–34 nucleotide length were size-selected by gel electrophoresis and later dephosphorylated by T4 Polynucleotide kinase (NEB). Ligation-free ribosome profiling libraries were generated from dephosphorylated footprints using the SMARTer small RNA-Seq Library Preparation Kit (Clontech) according to the manufacturer’s instructions^7^. rRNA depletion was performed by using the oligo pool^8^ annealing to rRNAs. The rRNA-depleted libraries were amplified by PCR. The libraries were quantified using a Qubit dsDNA HS kit (Thermo Fisher Scientific), and the library quality was assessed by a Bioanalyzer (Agilent). Sequencing of ribosome profiling libraries was performed using an Illumina NextSeq500 desktop sequencer with a read length of 50 bases.

### LC-MS/MS

Lysates were cleared by centrifugation at max for 15 min at room temperature. The supernatant was collected, and protein concentration was measured using the Pierce™ BCA protein assay kit (Thermo Fisher Scientific) following the manufacturer’s instructions. An equal amount of protein for each sample was first reduced with 5 mM Dithiothreitol (DTT) for 45 min at 25 °C with gentle mixing. Reduced samples were then alkylated with 10 mM Iodoacetamide (IAA) for 45 min at 25 °C with gentle mixing in the dark. Samples were later diluted in a 1:5 ratio with 50 mM Tris (pH 8.0) to decrease urea concentration below 2 M and digested with trypsin (Promega) in an enzyme-to-substrate ratio of 1:50 overnight at 25 °C with gentle mixing. After 16 hours of digestion, samples were acidified by adding 1% FA (final concentration) and desalted on in-house packed C18 StageTips as described previously^9^. Dried peptides by a vacuum concentrator were reconstituted with 3% acetonitrile and 0.2% formic acid for the subsequent liquid chromatography-tandem mass spectrometry (LC-MS/MS) analysis.

LC-MS/MS analysis was performed on a Q-Exactive HF. Approximately 0.5 μg of total peptides were analyzed on a Waters M-Class UPLC using an IonOpticks Aurora ultimate column (1.7 μm, 75 μm × 25 cm) coupled to a benchtop ThermoFisher Scientific Orbitrap Q Exactive HF mass spectrometer. Peptides were separated at a flow rate of 400 nL/min with a linear 95 min gradient from 2% to 22% solvent B (100% acetonitrile, 0.1% formic acid), followed by a linear 20 min gradient from 22% to 30% solvent B (100% acetonitrile, 0.1% formic acid), followed by a linear 9 min gradient from 30% to 60% solvent B (100% acetonitrile, 0.1% formic acid), followed by 36 min of wash and column equilibration. Each sample was run for 160 min, including sample loading and column equilibration times. The samples were measured in a Data Independent Acquisition (DIA) mode. MS1 Spectra were measured with a resolution of 120,000, an AGC target of 5e6 and a mass range from 350 to 1650 m/z. 47 isolation windows of 28 m/z were measured at a resolution of 30,000, an AGC target of 3e6, normalized collision energies of 22.5, 25, 27.5, and a fixed first mass of 200 m/z.

### Flow cytometry

Embryoid bodies (EBs) were collected and allowed to settle for 5–10 min at room temperature (RT), after which the media was removed. A single-cell suspension was obtained by treating the EBs with TrypLE Express (Fisher Scientific) supplemented with 50 μg/mL DNase (Calbiochem/Millipore), sufficient to fully immerse the EBs, for 15 min at 37 °C. Following centrifugation, the supernatant was removed, and the dissociated cells were fixed in 4% paraformaldehyde (PFA; Santa Cruz Biotechnology) for 15 min at RT, then washed twice with 1 × PBS (Fisher Scientific). Cells were permeabilized with 0.5% BSA–1% Triton X-100 (Fisher Scientific) for 5 min at RT. The primary antibody against cardiac troponin T (α-cTnT; Lab Vision, cat# MS-295-P1, RRID:AB_61808) was added at a 1:200 dilution and incubated for 20 min at RT. Cells were then washed twice with 0.5% BSA–1% Triton X-100 and incubated with the secondary antibody, goat anti-mouse IgG1 FITC (Santa Cruz Biotechnology, cat# sc2078), for 20 min at RT. After two additional washes in 0.5% BSA–1% Triton X-100, cells were filtered through a 40 μm cell strainer and analyzed using a FACS sorter (S3e, Bio-Rad) or FACS analyzer (NovoCyte, Agilent).

## Data Analysis

### RNA-seq and ribosome profiling

#### Annotation

A BED file containing all transcripts in the hg38 human reference genome was downloaded from Ensembl (version 32). The transcript set was then filtered using a custom Python script, which was built predominantly using tools from the plastid package. Only transcripts with an annotated coding sequence were retained. Duplicates and transcripts from unlocalized contigs were excluded from subsequent analysis. Transcript to gene name mappings were compiled from Ensembl using custom R code built using the biomaRt package. For RNA-seq alignment using STAR^10^, the final transcript set was exported as a GTF2 file using a custom Python script and used to construct a STAR index using the genomeGenerate command and specifying--sjdbOverhang 74. For Ribo-seq alignments, a separate STAR index was constructed using the same GTF2 file but specifying --sjdbOverhang 29. The GTF2 file was also used to construct the metagene annotation files used by programs in the plastid package. This was accomplished using the plastid metagene generate program and the flags --landmark cds_start and --annotation_format GTF2.

#### Read processing and alignment

RNA-seq reads were aligned to the genome using STAR. Unprocessed reads were fed to STAR using the--runMode alignReads--outSAMtype BAM SortedByCoordinate--outFilterType BySJout --outFilterIntronMotifs RemoveNoncanonicalUnannotated--outSAMstrandField intronMotif and--outFilterMultimapNmax 10 flags. Downstream tools to combine RNA-seq and Ribo-seq data were built to use single-end data, so the sense read of every aligned pair was extracted using samtools view (-b -q 30 -f 129), and the resulting bam file was used for downstream analysis.

For Ribo-seq, reads were pre-processed using cutadapt (v1.17) to remove the first three nucleotides and the poly-A tail (--cut 3 -a “A{15}” -j 8--nextseq-trim = 20--minimum-length 17). Bowtie (v1.2.2) was used to align reads to the human 45S pre-rRNA (NR_146144.1, downloaded from GenBank) using the -v 0 and--un flags to remove rRNA reads from the analysis. Reads were aligned to the genome using STAR (--runMode alignReads--outSAMtype BAM SortedByCoordinate --alignSJoverhangMin 400 --outFilterMismatchNmax 0 --outFilterMatchNmin 15 --outFilterMultimapNmax 1 --quantMode TranscriptomeSAM). Post-processing was performed using tools from the plastid package. The plastid peptidyl-site (P-site) program was used to estimate the location of ribosomal P-sites for each read length in each library (--min_counts 50 --require_upstream --min_length 17 --max_length 35). P offsets were generally in the range of 6–13, and some manual corrections were necessary in cases where the P-site program identified an incorrect peak. All manual corrections were done blinded to sample library identity. A default of 13 was used in cases where the P-site could not be clearly determined. For all subsequent analyses, reads were reduced to single counts offset from the 5′ end of the read by the value computed by the P-site program.

#### Read counting

Read counting of the RNA-seq and Ribo-seq data was performed using genomic alignments and a custom Python script built using tools from the plastid package. Briefly, for each gene, all associated transcripts were combined to create a single “meta-transcript”. Any genomic position mapping to the CDS of any annotated transcript was assigned to the CDS of the meta-transcript. Conversely, the 5′ - and 3′ UTRs of the meta-transcript were comprised of genomic positions assigned to the 5′/3′ UTR of at least one transcript but never to a CDS. Ribo-seq reads (reduced to a single count with a 5′ offset as defined above) in each defined meta region (5′ UTR, CDS, 3′ UTR) were counted. RNA-seq reads (reduced to a single count at the 5′ end) were counted in the meta-CDS and for the full meta-transcript. Count matrices were read into R and CDS counts for RNA-seq, and Ribo-seq were propagated forward into DESeq2^11^ using the DESeqDataSetFromMatrix function. CDS counts from genome-aligned libraries were normalized using DESeq2’s median of ratios method.

### Proteomics: Search and quantification

Proteomics raw data were analyzed using the library-based DIA search method (utilizing both DDA- and direct DIA-generated libraries) on SpectroNaut (v20.0) using a human UniProt database (Homo sapiens, UP000005640), under BSG factory settings, with automatic cross-run median normalization and imputation. Protein group data were exported for subsequent analysis.

### GO term enrichment analysis

Gene ontology enrichment analysis was conducted using the WebGestalt^12^ (http://www.webgestalt.org) platform using the Gene Set Enrichment Analysis (GSEA)^13^ on biological processes non redundant terms.

### Principal component analysis and batch correction

To account for batch effects between biological replicates, we applied the removeBatchEffect function from the limma R package (v3.60.6)^14^. Batch-corrected, log2-transformed expression data were then used to perform principal component analysis (PCA) using the prcomp() function in R (v4.4.0). PCA plots were generated based on the first two principal components to visualize sample clustering and temporal trajectories.

## Data Records

RNA-seq and ribosome profiling datasets have been deposited in the NCBI GEO database under accession numbers GSE274620^15^ and GSE274622^16^, respectively. The mass spectrometry data are available in the MassIVE repository with the accession number MSV000095595^17^.

The RNA-seq, ribosome profiling, and proteomics datasets include both raw data and processed quantification files, deposited in the repositories mentioned above. The raw RNA-seq and ribosome profiling files are labeled to indicate the data type, biological replicate, and time point of collection during cardiomyocyte differentiation. Processed gene-level count matrices are provided for both RNA-seq and ribosome profiling, in normalized and raw formats. Columns in these tables represent samples from two biological replicates collected across multiple time points, with column names reflecting replicate and day identifiers. The mass spectrometry dataset includes raw LC-MS/MS data acquired in DIA mode, similarly labeled by replicate and time point. Protein group quantification tables are included with and without imputation, containing intensity values for identified protein groups across time-course samples. All quantification files are organized in a wide format, with rows representing genes or proteins and columns corresponding to replicate-day combinations.

## Technical Validation

### Cardiomyocyte differentiation efficiency

**Fig. 2:**
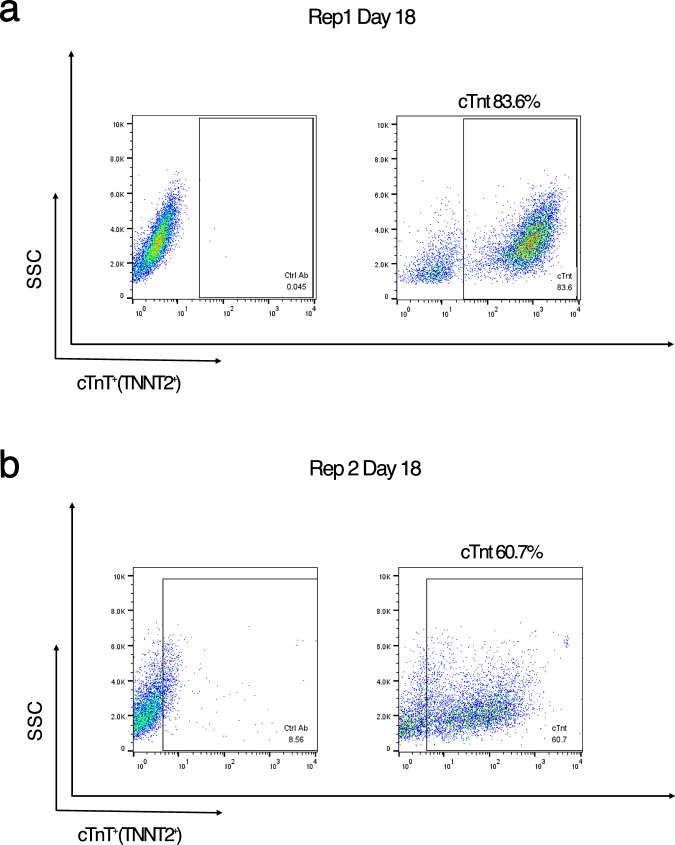
Quantification of cardiomyocyte differentiation efficiency by flow cytometry. (**a**) Replicate 1 and (**b**) Replicate 2: Flow cytometry analysis of cardiac troponin T (cTnT; *TNNT2*) expression at day 18 of differentiation. Cells were stained with an anti-cTnT primary antibody and analyzed by FACS. Left panels show negative controls stained with secondary antibody only; right panels show specific staining with the cTnT antibody. The percentage of cTnT^+^ cells was 83.6% in replicate 1 and 60.7% in replicate 2.

To assess the efficiency of cardiomyocyte differentiation, we performed flow cytometry analysis on day 18 of differentiation using cardiac troponin T (*TNNT2*) as a marker. The proportion of cTnT^+^ cells was approximately 84% in replicate 1 (Rep1) and 61% in replicate 2 (Rep2) (Fig. 2a,b), indicating robust cardiomyocyte differentiation. Notably, we did not apply lactate purification in this study, as our goal was to capture native gene expression dynamics without perturbation. Lactate-based selection, used in certain protocols to enrich cardiomyocytes, has been shown to induce transcriptional changes that could confound developmental trajectories^18^. Our approach provides an unaltered view of cardiomyocyte differentiation while still achieving high efficiency, supporting the utility of this dataset for studying endogenous gene regulation during development.

### Validation of biological consistency

**Fig. 3:**
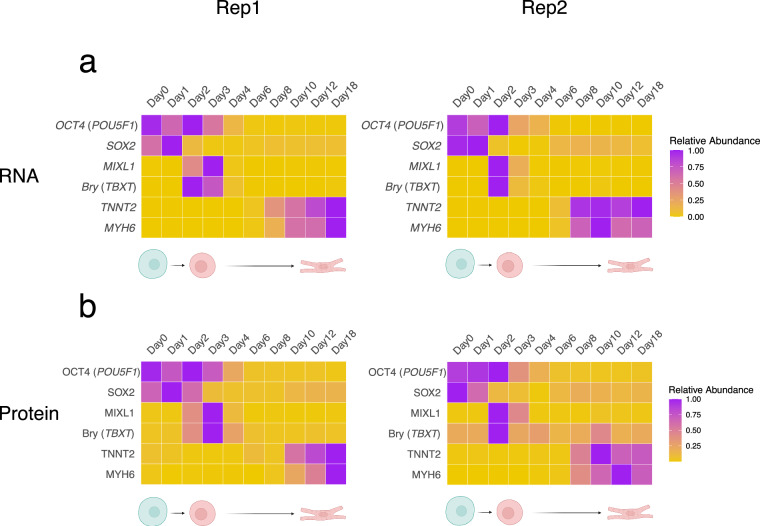
Expression of differentiation markers at (**a**) RNA and (**b**) protein levels during hESC to cardiomyocyte differentiation for both replicates. *OCT4* (*POU5F1*) and *SOX2*, hESC markers; *MIXL1* and Brachyury (*TBXT*), mesoderm markers; Troponin T2 (*TNNT2*) and *MYH6*, cardiomyocyte markers. The color scale represents normalized expression values (expression levels normalized to the maximum expression value of each gene across all time points), with purple indicating high expression and yellow indicating low expression.

**Fig. 4:**
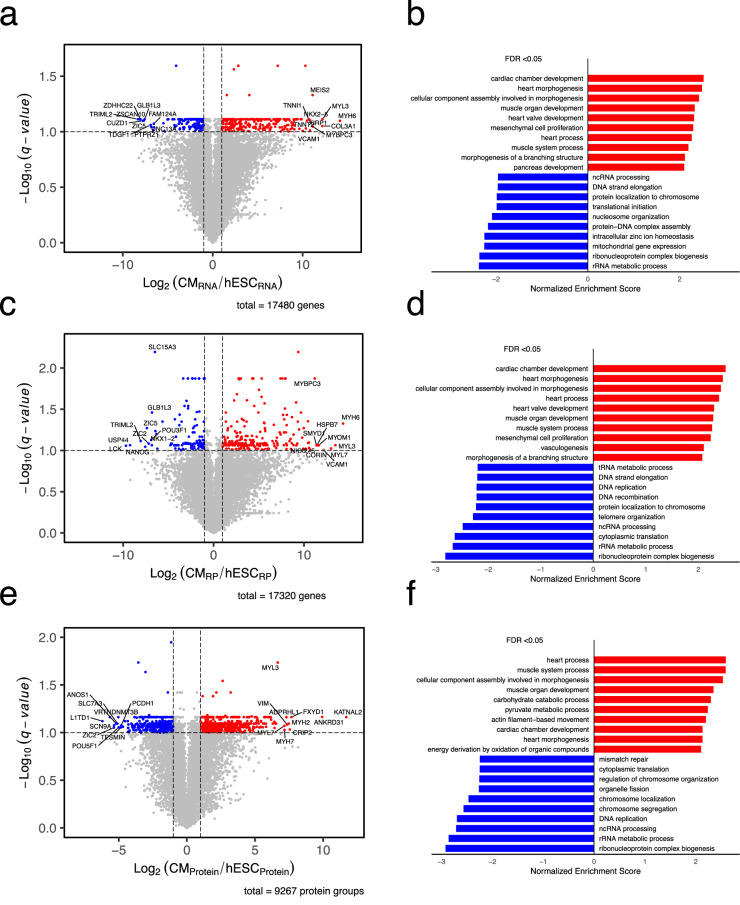
Comparison of cardiomyocyte to hESC expression at RNA, translation, and protein levels. (**a,c,e**) Volcano plots comparing gene expression levels between cardiomyocytes and hESCs at the (**a**) RNA, (**c**) translation, and (**e**) protein levels, respectively. The x-axis represents the log₂ fold change of gene expression (Cardiomyocyte/hESC), and the y-axis represents the –log₁₀ of the adjusted p-value (q-value), calculated using two-tailed Student’s t-tests followed by Benjamini-Hochberg correction. Genes significantly upregulated in cardiomyocytes (log₂(FC) > 1 and q < 0.1) are highlighted in red, and genes significantly downregulated (log₂(FC) < –1 and q < 0.1) are highlighted in blue. The top 10 genes with the strongest increase and decrease in fold change that meet the q-value cutoff are labeled with their gene IDs. CM = Cardiomyocyte, RP = Ribosome profiling. (**b,d,f**) GSEA performed on genes ranked by their fold change in the (**b**) RNA, (**d**) translation, and (**f**) protein levels, respectively, revealed an enrichment of Gene Ontology (GOs) terms associated with cardiomyocyte differentiation and function, and a depletion of GO terms related to DNA replication and ribosome biogenesis. GO terms shown passed an FDR threshold of < 0.05.

To assess biological consistency, we confirmed the expression of differentiation markers at both the mRNA and protein levels. Human embryonic stem cells (hESCs) expressed the pluripotency markers *OCT4* (*POU5F1*) and *SOX2*. Mesodermal cells were validated by the expression of the known markers *MIXL1* and Brachyury (*TBXT*), while cardiomyocytes were confirmed through the expression of the cardiomyocyte-specific markers, such as Troponin T2 (*TNNT2*) and *MYH6* (Fig. 3a,b). Notably, there was a slight delay in cardiomyocyte differentiation in replicate 1 relative to replicate 2, as evidenced by the expression patterns of these markers. Furthermore, differentially expressed genes between cardiomyocytes and hESCs were identified using RNA-seq, Ribo-seq, and LC-MS/MS data (Fig. 4a,c,e). Gene Ontology (GO) analysis, utilizing GSEA with genes ranked by their fold change, revealed a significant enrichment of GO terms associated with cardiomyocyte differentiation and function, while showing a depletion of GO terms related to DNA replication and ribosome biogenesis (Fig. 4b,d,f).

### Validation of dataset reproducibility

**Fig. 5:**
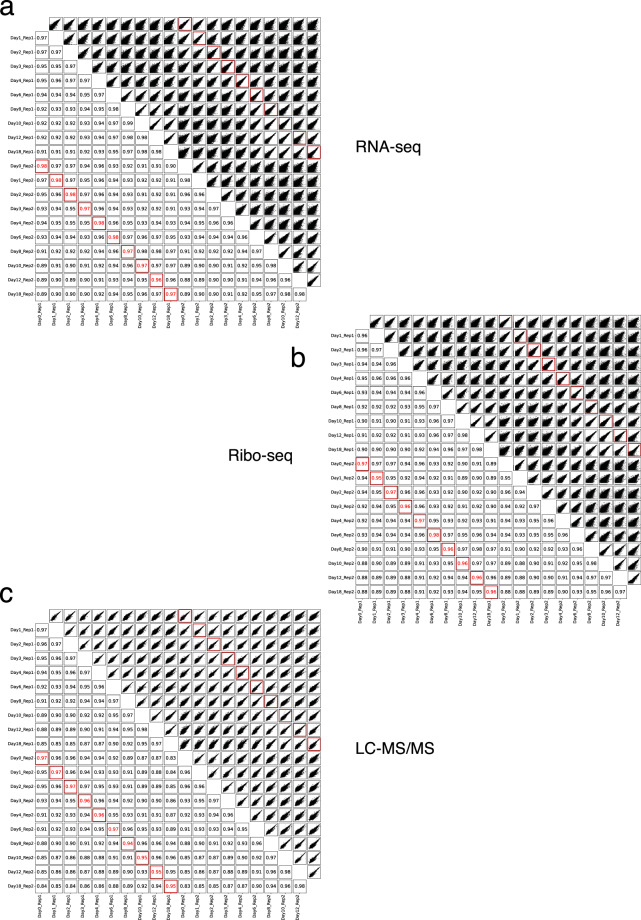
Correlation analysis of samples across all time points and replicates for each omics layer. (**a**) RNA-seq, (**b**) Ribo-seq, and (**c**) LC-MS/MS. Each panel shows all pairwise comparisons between samples. The upper right triangle displays scatter plots, and the lower left triangle shows Pearson correlation coefficients (R) for each pair. Biological replicate pairs from the same time point are highlighted in red: both their scatter plots (upper right) and correlation values (lower left) are enclosed in red boxes.

To assess reproducibility and temporal relationships across the dataset, we computed pairwise Pearson correlations between all samples for RNA-seq, Ribo-seq, and LC-MS/MS data (Fig. 5a–c). Biological replicates from each time point showed high reproducibility across all data types, with correlation coefficients ranging from 0.96 to 0.98 for RNA-seq, 0.95 to 0.98 for Ribo-seq, and 0.94 to 0.97 for proteomics. Neighboring time points, both within and across replicates, also exhibited strong correlations, consistent with gradual and coordinated transitions in gene expression during differentiation. Notably, at midpoints in the time course, replicate 1 appeared slightly delayed compared to replicate 2; this was reflected in slightly stronger correlations between replicate 1 samples and the preceding time point of replicate 2 (e.g., Rep1 Day 10 and Rep2 Day 8).

### QC on the RNA-seq data

**Fig. 6:**
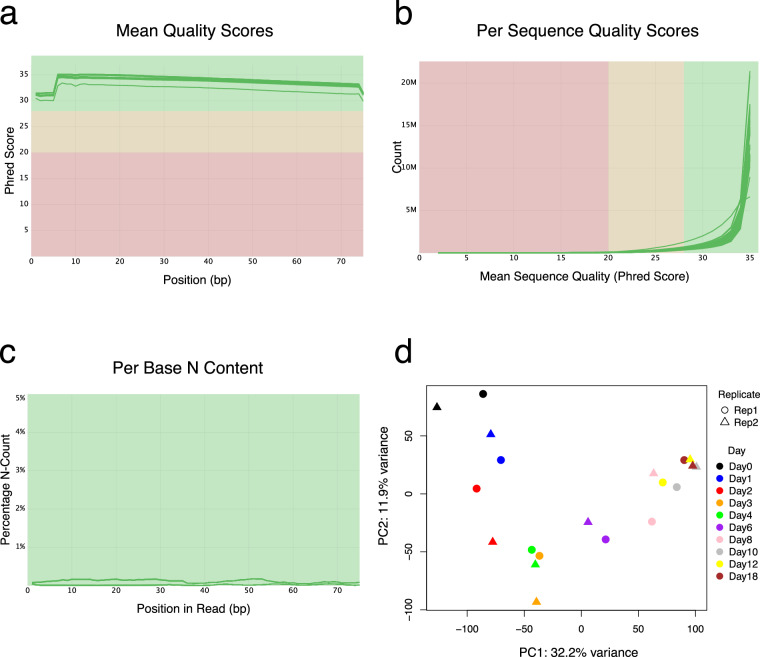
Quality control and PCA of the RNA-seq data. (**a**) Mean quality scores for each base position in the reads, presented as Phred scores for all samples. (**b**) Distribution of quality scores across all reads obtained for all samples. (**c**) Percentage of undetermined bases (N) at each position along the read. Individual samples are denoted by the green lines. The green shaded area indicates high-quality scores, the yellow area indicates moderate quality, and the red area indicates low quality. (**d**) Principal Component Analysis (PCA) illustrating the variance in RNA expression data across different days of differentiation and replicates.

Quality control was performed on all RNAseq samples using FastQC^19^, with sequencing depth ranging from 20 to 35 million paired-end reads per sample and approximately 40% unique reads for each sample. The FastQC results were then merged using MultiQC^20^ and high-quality was observed across all samples, including metrics such as mean quality scores (Fig. 6a), per sequence quality scores (Fig. 6b), and per base N content (Fig. 6c). After alignment and transcript quantification, Principal Component Analysis (PCA) was conducted to evaluate the variance in gene expression data across different days of differentiation (Fig. 6d). The variance is clearly delineated over the course of differentiation, with distinct separation of samples according to their respective days. Replicates from the same time point generally cluster closely, reflecting high overall reproducibility and consistent temporal progression in the differentiation process. This separation along the principal components highlights the dynamic changes in gene expression as hESCs differentiate into cardiomyocytes over time.

### QC on the Ribo-seq data

**Fig. 7:**
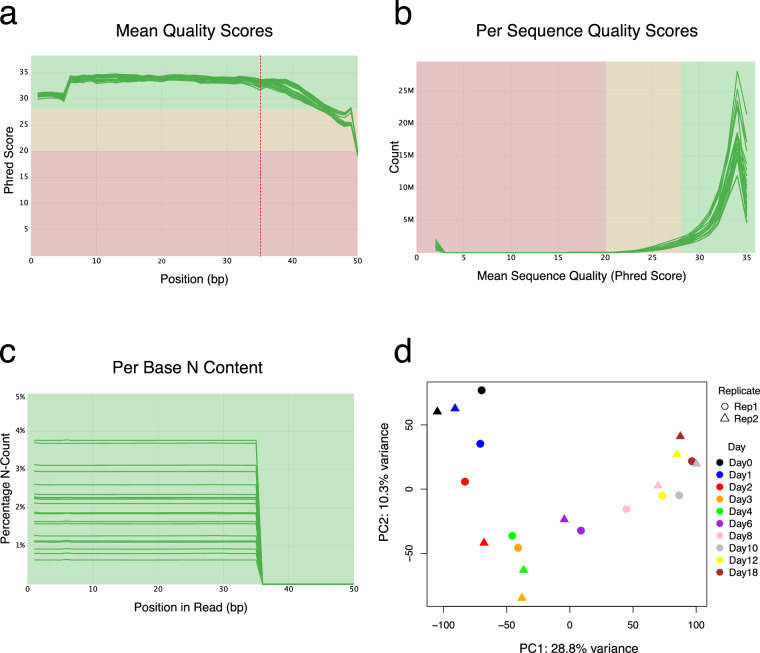
Quality control and PCA of the Ribo-seq data. (**a**) Mean quality scores for each base position in the reads, presented as Phred scores for all samples. The red dashed line indicates the start of the poly(A) tail. (**b**) Distribution of quality scores across all reads obtained for all samples. (**c**) Percentage of undetermined bases (N) at each position along the read. Individual samples are denoted by the green lines. The green shaded area indicates high-quality scores, the yellow area indicates moderate quality, and the red area indicates low quality. (**d**) Principal Component Analysis (PCA) illustrating the variance in translation (Ribo-seq) data across different days of differentiation and replicates.

The quality of raw sequencing data was assessed using FastQC. Sequencing depth ranged from 40 to 90 million single-end reads per sample, with approximately 20% unique reads per sample. A decrease in the mean quality score was noted towards the end of the 50 bp ribosome footprints, attributed to the poly(A) tail added during library preparation for cDNA synthesis (Fig. 7a). In all other cases, high-quality metrics were consistently observed (Fig. 7b,c). PCA of the Ribo-seq data similarly revealed structured variance across the time course, with replicates from the same day exhibiting similar profiles, indicating high reproducibility and temporal consistency. (Fig. 7d).

**Fig. 8:**
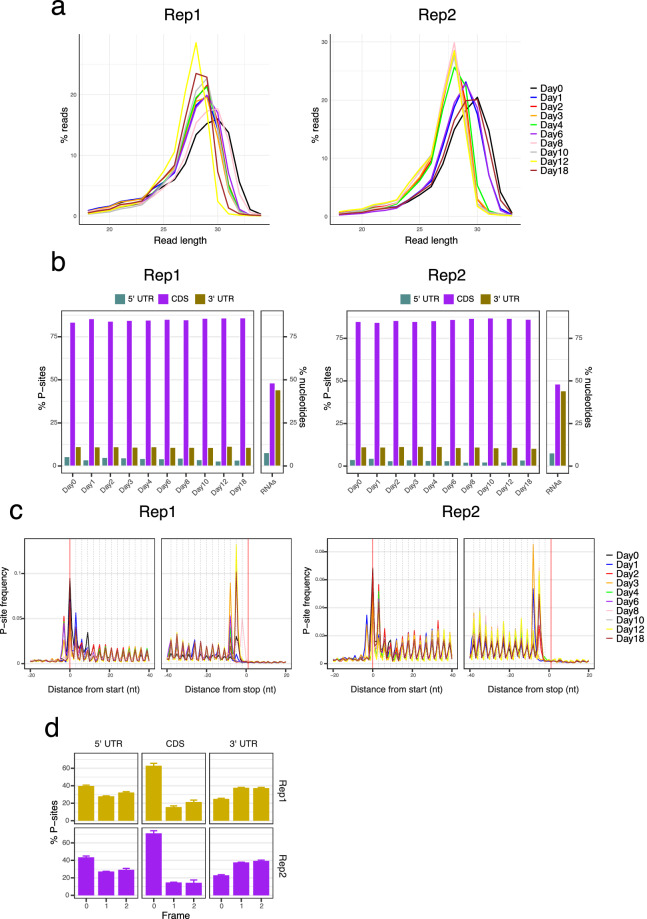
Diagnostic analysis of the Ribo-seq data. (**a**) Read length distribution of ribosome-protected fragments across different days of differentiation for replicate 1 and replicate 2. (**b**) Distribution of peptidyl-sites (P-sites) across different regions of transcripts (5′ UTR, CDS, and 3′ UTR) for each day of differentiation in Rep1 and Rep2. The bar labeled as “RNAs” displays the expected read distribution from a random fragmentation of RNA. (**c**) P-site frequency distribution relative to the start and stop codons across different days of differentiation in Rep1 and Rep2. (**d**) Distribution of P-sites across the three nucleotide frames (0, 1, and 2) within different transcript regions (5’ UTR, CDS, and 3’ UTR) for Rep1 and Rep2. The panels show the average taken across all time points in each replicate.

To verify that the ribosome profiling data accurately reflects the underlying biology and is free from technical biases or errors that could affect downstream analyses, diagnostic analyses were performed using riboWaltz^21^ (version 2.0). The read length distribution for both replicates demonstrated ribosome-protected fragments predominantly around the expected size of 28–30 nucleotides (Fig. 8a). Moreover, the ribosome profiling reads primarily aligned to the coding sequences (CDS) of transcripts, which exhibited the highest percentage of reads in both replicates (Fig. 8b). A key feature of ribosome profiling data is the trinucleotide periodicity of ribosome footprints along the coding sequences due to the ribosome movement from one codon to the next. Our analysis demonstrated that the identified peptidyl-sites (P-sites) at each time point in each replicate exhibit this codon periodicity (Fig. 8c). In line with this result, the distribution of P-sites across the three nucleotide frames (0, 1, and 2) reveals a strong trinucleotide periodicity within the CDS region, predominantly aligning to frame 0 in both replicates (Fig. 8d). This consistent alignment within the CDS, along with the more even distribution in the 5′ and 3′ UTRs, confirms the accuracy and reliability of our ribosome profiling data.

### QC on the LC-MS/MS data

**Fig. 9:**
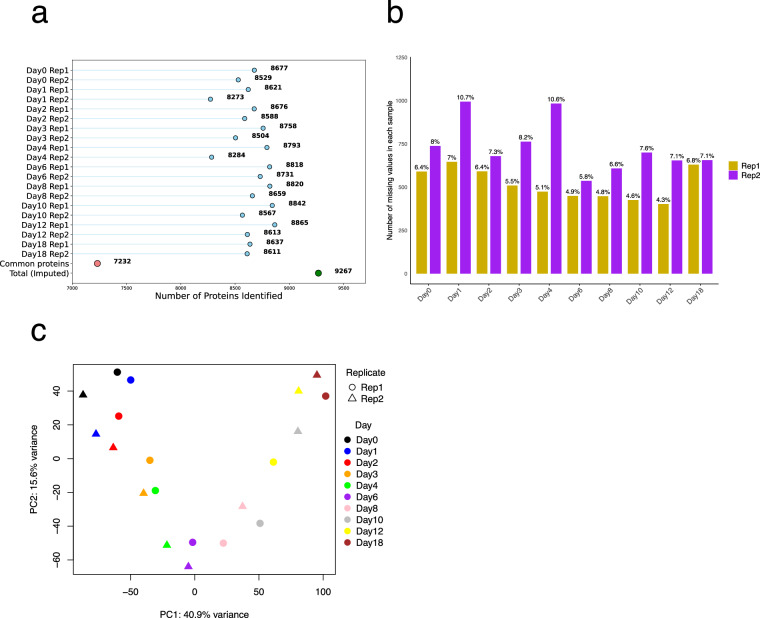
Quality control and PCA of the LC-MS/MS data. (**a**) Number of protein groups identified across different days of differentiation for replicate 1 and replicate 2. Blue dots represent the number of protein groups identified in each specific sample without imputation. The red dot indicates the 7232 protein groups commonly identified across all samples, while the green dot indicates the total number of protein groups identified with imputation (9267). (**b**) Percent and number of missing values in protein group identifications across different days of differentiation for Rep1 and Rep2. (**c**) Principal Component Analysis (PCA) illustrating the variance in protein expression data across different days of differentiation and replicates.

Protein expression data were collected by data independent acquisition (DIA) LC-MS/MS. The protein group output generated by SpectroNaut with cross-run median normalization and global imputation was utilized for all analyses. However, data completeness was specifically assessed in this section. A total of 9,267 protein groups were identified across all time points with imputation of missing values for each sample, while an average of 8,640 protein groups per sample were identified without imputation (Fig. 9a). Of these, 7,232 protein groups were consistently identified in all samples (Fig. 9a), with an average missing rate of 6.7% (Fig. 9b). Sample variance was evident by PCA, with clear separation of samples according to their respective days in the differentiation process. Replicates from most time points formed tight clusters, reflecting high overall reproducibility and consistency (Fig. 9c).

## Data Availability

Pre-processing scripts for RNA-seq and ribosome profiling datasets are available in the GitHub repository (https://github.com/ak3952/scientific_data_hESCtoCardiomyocyteDifferentiation).
